# Moderating role of CEO expertise on the relationship between capital structure and financial reporting timeliness of Saudi-listed companies

**DOI:** 10.1371/journal.pone.0338840

**Published:** 2026-03-06

**Authors:** Hamid Ghazi H Sulimany, Ehsan Almoataz, Adnan Ali, Faisal Faisal, Abdulrahman Atllah Alharbi

**Affiliations:** 1 Accounting Department, Business Administration College, Taif University, Ta’if, Saudi Arabia; 2 Accounting Department, College of Business and Economics, Umm Al-Qura University, Makkah, Saudi Arabia; 3 Faculty of Business, Sohar University, Oman; 4 School of Accounting, Faculty of Business & Management, University Sultan Zainal Abidin, Kuala Nerus, Terengganu Darul Iman, Malaysia; 5 Altinbas Cyprus University, Nicosia/TRNC, Mersin, Turkey; 6 Department of Accounting and Finance, Institute of Business Studies and Leadership, Faculty of Business and Economics, Abdul Wali Khan University, Mardan, KP, Pakistan; Liverpool John Moores University, UNITED KINGDOM OF GREAT BRITAIN AND NORTHERN IRELAND

## Abstract

In this study, we investigated the effect of capital structure on financial reporting timeliness with an interaction role of CEO financial expertise. Using the fixed effects technique, we analysed data from listed firms on the Saudi Stock Market between 2014 and 2023. Our results showed that capital structure choice through debt financing may significantly influence firms to reveal their audited accounts on a timely basis to signal their financial capabilities. Additionally, the results provide strong evidence that a CEO’s financial expertise may enhance the role of debt financing in reducing audit report delays, consistent with upper echelons and agency theories. The findings appear to be robust with the use of alternative measures, the COVID-19 effect and endogeneity control.

## 1. Introduction

The primary objective of financial reporting is to provide financial information to investors and other relevant stakeholders to enable them to meet their diverse needs [1–3]. This information can only be useful in making such decisions if it is released in a timely manner. In this context, timeliness refers to the process of releasing year-end financial statements as soon as they have been certified by external auditors [4,5]. In light of the above information, companies should take measures to ensure a shorter audit report lag to facilitate the timely release of audited accounts. Within several accounting standards and frameworks, financial timeliness has been recognised as integral to financial reporting quality [6–8]. In these frameworks, it is emphasised that, if financial statements are not reported promptly, the information therein may lose relevance and make it less valuable for use in decision-making [9–11]. In the literature, financial reporting timeliness is considered a crucial mechanism for reducing information asymmetry between company management and other relevant stakeholders [12–14]. Bridging this gap can help lower agency conflicts in companies, foster accountability and boost investors’ confidence.

Drawing from the agency framework and the upper echelons perspective, capital structure is associated with the timeliness of financial reporting. In the literature, it is argued that capital structure decisions using debt financing serve as a crucial strategy for enhancing managerial efficiency [15,16]. In particular, researchers have reported that borrowings may stimulate firms’ managers to embrace timely financial disclosure to signal their commitment to debt servicing and raise creditors’ confidence [6,17,18]. This conclusion suggests a positive link between capital structure choices and timely financial reporting. In contrast, in one segment of the literature, a negative relationship has been noted between debt financing and the timeliness of financial reports [1,3,5,8,10]. The results of these studies have emphasised that firms with substantial debt may delay the release of financial reports because a high debt ratio may signal the likelihood of bankruptcy. From the above, it is evident that contrary views are documented in the literature concerning the nexus between capital structure and financial reporting timeliness. This inconsistent empirical finding stands as the motivating factor in conducting this research. In this study, our aim is to answer the following question: When does capital structure influence financial reporting timeliness? We aim to fill the aforementioned gap in the literature by advocating the potential of CEO financial expertise in ensuring timelier financial disclosure, thereby advancing the empirical literature.

It is widely emphasised that top managers’ attributes, such as educational background, may influence firms’ strategic choices and outcomes [19,20]. In numerous studies, researchers have appraised financial expertise’s potential in shaping organisational efficiency and remediating internal control lapses [21–23]. In addition, results presented in the literature suggest that firms managed by CEOs with financial knowledge are associated with a lower incidence of earnings manipulation and have robust internal governance. CEOs with accounting and finance knowledge may focus greater attention on the actions of the internal audit department to ensure that the unit performs its functions diligently [21,24]. Given the vital role of CEO financial expertise in shaping organisational efficiency, we aim to investigate the moderating effect of CEO financial expertise on the link between capital structure and financial reporting timeliness using a sample of Saudi-listed companies from 2014 to 2023.

Through these efforts, we supplement the current literature in the following manner. Our findings contribute to the growing body of literature on the relationship between capital structure and financial reporting timeliness. Our findings reinforce the strategic influence of debt financing in promoting timely financial disclosure to meet investors’ needs and increase their confidence. Secondly, we offer a unique perspective on the potential of CEO financial expertise to strengthen the monitoring role of debt financing, thereby reducing information disparity between firms and investors. This evidence unveils a fresh view of the determinants of timelier financial reporting, thus providing insight into improving managerial efficiency and mitigating agency conflicts in organisations. We conducted additional analyses using the generalised method of moments (GMM) framework. The results enable us to provide a more robust outcome that addresses simultaneity and endogeneity. The research findings may also have certain policy and regulatory implications for corporate disclosure. The findings highlight to regulators and corporate owners the importance of CEO financial expertise in enhancing firms’ internal audit units and promoting financial reporting quality.

In the remainder of this paper, we present the Saudi context, the literature review and our methodology. We also present the research findings and the subsequent discussion, followed by robust analysis. In the final paragraph, we focus on the concluding remarks.

## 2. The Saudi context

In this study, we focus on Saudi Arabia because of the country’s unique institutional setting, with corresponding implications regarding corporate disclosure [8,25]. The nation is rich in crude oil, and thus, its corporate governance system may be of interest to investors globally [10,26]. However, the corporate sector is characterised by a high level of information asymmetry due to underdeveloped markets for corporate control. A crucial feature of Saudi Arabia is the fact that family and royal ownership dominate the country’s corporate sector [27,28]. These factors may promote managerial entrenchment, leading to high agency conflicts and weakening corporate governance practices. The country emphasises Islamic principles, and corporate bodies tend to attach less emphasis to formal accountability mechanisms when making decisions [29,30]. This practice greatly influences regulatory adherence and enforcement.

However, the Saudi authorities have made substantial efforts to strengthen compliance with various corporate governance codes (CGC) to foster strong disclosure and accountability. Such efforts led to the enactment of the country’s CGC in 2017 to boost capital market performance and investors’ confidence [3,31]. Likewise, several reforms, such as Vision 2030, were designed to open the economy to foreign investment to enhance the country’s gross domestic product [32,33]. Findings presented in the literature have revealed the potential of CEO financial expertise in shaping corporate disclosure and accountability [21–24]. Hence, CEO financial expertise may represent an important mechanism in the Saudi corporate sector. The effectiveness of financial knowledge can neutralise the potential agency issues emanating from the strong influence of family ownership. This improvement may help make corporate boards more independent, enhance internal control and ensure adherence to formal accountability mechanisms. Empirical analysis on strengthening corporate disclosure and financial reporting quality may therefore be of interest to policymakers and regulators to enable them to meet the needs of diverse investors.

## 3. Literature review

### 3.1. Theories

The framework of agency theory can be used to describe the background of the relationship between capital structure and financial reporting timeliness. Based on this theory, it is postulated that corporate organisations may suffer from agency conflicts and high information asymmetry because of the separation between management and capital providers [34,35]. Within this framework, it is argued that managers may make decisions that maximise their interests. It is concluded that managers must be monitored through various corporate governance mechanisms to ensure greater disclosure and accountability [36–38]. One such control tool advocated by this theory is firms’ capital structure choices. Within this perspective, it is argued that firms should design their capital structures with higher debt to enhance managerial efficiency, such as the timely disclosure of financial reports [8,39]. Debt financing may therefore enhance financial reporting timeliness due to pressure from creditors. These creditors require such information to make their investment decisions. Hence, this perspective emphasises a positive association between capital structure and financial reporting timeliness. In comparison, agency theory may also underpin the nexus between CEO financial expertise and financial reporting timeliness. Through this theory, it is argued that the sophisticated knowledge of these CEOs may assist firms in preventing earnings management incidents and boosting financial reporting quality [7,40]. In the same context, the theory suggests that sound monitoring from CEOs with financial knowledge may enable firms to embrace timely financial disclosure [41,42].

From a different perspective, the upper echelons theory can be leveraged to support this study. Based on upper echelons theory, organisations can significantly benefit from the managers’ competence, skills and technical knowledge because their attributes are crucial governance drivers [19,23]. The proponents of this theory also argue that a CEO with financial knowledge may use their professional training to formulate sound decisions that can enhance corporate disclosure [41,43]. Greater disclosure due to the managerial skills and leadership style of financial experts may strengthen internal audit functions and minimise audit queries. Hence, within this framework, it is assumed that firms with CEOs who are financial experts are more likely to provide more rapid disclosure of audited accounts. In addition, based on this theory, it is posited that a CEO’s financial expertise can significantly influence corporate strategic decisions and outcomes [44,45]. Hence, CEOs with financial knowledge can leverage their expertise to design sound capital structures and streamline financial reporting systems.

Signalling theory can also be employed in this context. Based on this theory, it is argued that organisations engage in specific behaviours or actions to convey useful information to others [46,47]. As part of this theory, it is suggested that conveying credible information to stakeholders and the external environment helps reduce information asymmetry. Disclosure of a firm’s competence or prospects signals its governance quality to diverse stakeholders and boosts investors’ confidence. Disseminating positive news enables a firm to gain a competitive advantage and attract more investment opportunities [48,49]. Useful information includes stakeholders, including capital structure decisions, financial indicators and other outcomes. Signalling theory is relevant in this discourse because capital structure composition signals information about a firm’s prospects. Specifically, debt commitment reveals a firm’s creditworthiness and financial stability to creditors and other stakeholders [50,51]. This theory suggests a positive relationship between capital structure and financial reporting timeliness because financial leverage may stimulate firms to adopt timely disclosure of audited accounts to signal their capability to repay debt in order to satisfy creditors’ demands [8,21].

### 3.2. Hypotheses formulation

**Fig 1:**
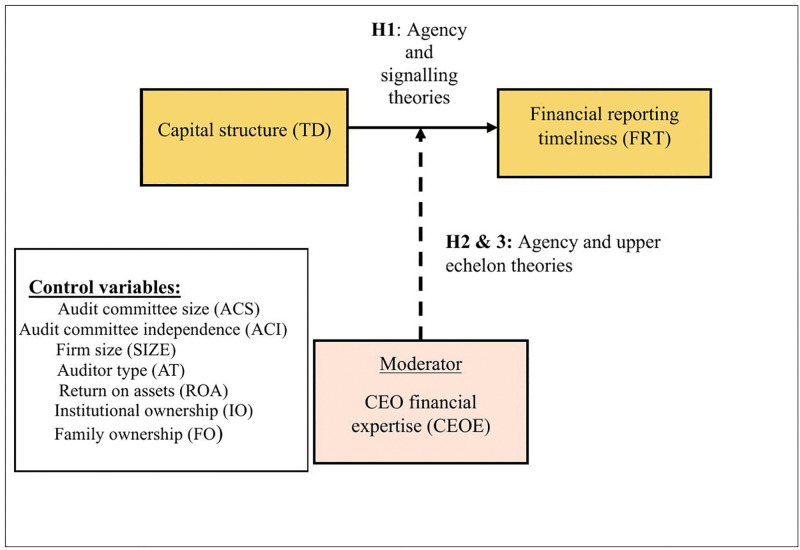
Research framework.

In this section, we present the development of the study’s hypotheses, which are illustrated in Fig 1, i.e., Research Framework.

#### 3.2.1. Capital structure and financial reporting timeliness.

In the literature, capital structure choices are considered a strategy for enhancing managerial efficiency and boosting firm performance [6–8]. In particular, researchers have extensively focused on the role of debt financing in influencing organisational outcomes. Accordingly, it has been stated that debt financing prompts managers to make a more concerted effort to generate sufficient cash flows to service the borrowings [3,21]. From a different perspective, capital providers such as creditors require audited financial reports to monitor fund utilisation [6,52]. Therefore, firms with high amounts of debt in their capital structure may attempt to meet creditors’ demands by disclosing financial information on a timely basis to retain creditors’ confidence [5,10]. Financing capital structure through debt is thus positively associated with the timeliness of financial reporting. Conversely, in some studies, it has been emphasised that high debt levels may impede timelier financial reporting disclosure [9,50]. Potential investors consider firms with substantial borrowings to be prone to bankruptcy and liquidation [10,53]. Firms with substantial borrowings may choose to delay disclosure of their financial statements to avoid revealing unfavourable information to the market and regulatory agencies [8,21]. In light of the above findings, the following hypothesis is formulated:

**H1:** Debt financing increases financial reporting timeliness.

#### 3.2.2. CEO financial expertise and financial reporting timeliness.

A chief executive officer (CEO) heads a company’s management team and initiates policies that can enhance the firm in different ways [41,54]. Financial expertise is a crucial mechanism that can assist the CEO in managing a firm’s resources efficiently, in light of their educational training [55–57]. It has been argued that CEOs with accounting and finance knowledge may pay greater attention to the actions of the internal audit department to ensure that the unit performs its functions efficiently [21,24]. Such efforts can facilitate the discovery and curbing of damaging practices that can weaken the internal control system. Financial expertise may enable the CEO to address complicated accounting issues and engage in meaningful discussions that may improve financial reporting quality [6,58]. Hence, it has been stated that firms run by CEOs with financial expertise tend to have more robust financial reporting systems due to their unique perspective and conservative financial policies [42,59]. Considering the points raised above, it has been argued that firms with CEOs with financial knowledge may be associated with sound internal governance systems and a lower number of audit queries, leading to the timely release of financial reports. In light of the above arguments, the following hypothesis is presented:

**H2**: CEO financial expertise may result in higher financial reporting timeliness.

#### 3.2.3. Moderating effect of CEO financial expertise.

In numerous studies, the importance of financial expertise in shaping board functions as been emphasised [32,55,60]. The CEO is the highest-ranking officer and is expected to develop policies and present them to the board for approval [54,61]. In this regard, it is suggested in the literature that the financial background of a CEO may positively influence corporate outcomes because their knowledge may guide management decisions [32,62]. Two theories are employed to explain the moderating role of CEO financial expertise. Following agency theory arguments, CEO financial expertise may promote board monitoring and decision-making capabilities due to greater financial management knowledge [21,63]. In other studies, it has been found that firms with CEOs who are financial experts are more likely to eliminate managerial inefficiencies and disclose financial records more frequently to the public [6,8]. The above measures may help minimise information disparity between firms and users of financial statements.

In comparison, the upper echelons theory may support the moderating role of CEO financial expertise. Within this framework, analyses are undertaken to determine how the cognitive capabilities of top managers may determine corporate outcomes [20,44]. As part of this theory, it is argued that financial expertise may facilitate sound decisions that enhance corporate disclosure [41,43]. Building on these views, it is assumed that CEO financial expertise may moderate the effect of capital structure on financial reporting timeliness. Based on the above findings, we present the following hypothesis:

**H3:** The effect of debt financing on financial reporting timeliness may be greater in firms with a financial expert as CEO.

## 4. Method

### 4.1. Sampling

The sample size covers 114 non-financial listed companies on the Tadawul (Saudi) Stock Exchange. The data of these companies from 2014 to 2023 was extracted from the Eikon database and the Tadawul website. The nature of this research suggests that we should utilise firm-level and corporate governance data. The corporate governance information was obtained from the Tadawul website comprising the selected companies listed on the Saudi stock market website. The Eikon Datastream was utilised to generate the firm variable data. The sampling procedure can be explained as follows: The total number of companies considered was 182, and 42 financial companies were excluded because of their unique capital structure compositions and financial reporting framework [8,64]. In this case, 420 observations from financial companies were removed. In addition, 260 observations were excluded for 26 companies with substantial missing data and those lacking debt capital in their financial statements. In this study, we measured capital structure using the debt ratio, consistent with the theoretical arguments [8,39]. This approach enables us to test the monitoring and signalling effects of leverage. Therefore, to avoid skewed analysis and enhance the consistency of the empirical results, firms without borrowings in their financial statements were excluded from the sample. The final sample comprised 1,140 firm-year observations (114 firms) from 12 sectors, as depicted in Table 1.

**Table 1:** Sample of the study.

Sector	No. of firms	%
Materials	31	27.19
Foods and beverages	16	14.04
Real estate	13	11.40
Capital goods	11	9.65
Transport	9	7.90
Health	7	6.14
Telecommunications	6	5.26
Consumer durable	4	3.51
Consumer services	6	5.26
Foods retailing	5	4.39
Retailing	3	2.63
Utilities	3	2.63
**Total**	**114**	**100**

### 4.2. Empirical model

We used regression analysis to investigate the relationship between capital structure and financial reporting timeliness, in addition to the moderating effect of CEO expertise. We employed a panel data approach, as the data collected comprises a time series from multiple firms. The panel data research method produces more efficient regression estimates because it offers large data points, reduces estimation bias and provides more freedom [60,65,66]. The Hausman test was applied to select the appropriate analytical tool between fixed and random effects. The Hausman test results revealed that the fixed effects estimation technique is more suitable for this work. Specifically, we employed a two-way fixed effects model to control for firms’ and time effects, thus capturing the heterogeneity of the sampled firms [65,67]. This procedure helps boost the efficiency of the panel data analysis. We thus developed the following empirical models using financial reporting timeliness as the dependent variable:


$${FRT}_{it}=\emptyset \;+\beta_{\text{1}}{TD}_{\text{it}}+\beta_{\text{2}}{ACS}_{\text{it}}+\;\beta_{\text{3}}{ACI}_{\text{it}}+\beta_{\text{4}}{SIZE}_{\text{it }}+\beta_{\text{5}}{AT}_{\text{it}}\;+\beta_{\text{6}}{ROA}_{\text{it}}+\beta_{\text{7}}{IO}_{\text{it}}+\beta_{\text{8}}{FO}_{\text{it}}+\mu_{i}{\;+\varepsilon }_{it}$$ (1)

Regarding the moderating influence of CEO expertise on the link between capital structure and financial reporting timeliness, we inserted the moderating variable (CEOE) and the interaction term (TD * CEOE) into (1) to determine their effects in the model. In the literature, a moderator is defined as a variable that influences the relationship between an explanatory variable and a dependent variable [68,69]. The criterion for moderation analysis is that moderation occurs when the interaction term (path c) is significant [69,70]. The specified moderation model is shown in (2), and the variables’ definitions are presented in Table 2.

**Table 2:** Measurement of the variables.

Variable	ACRONYM	Measurement
**Dependent variable:**		
Financial reporting timeliness	FRT	The number of days between the end of a firm’s accounting year and the external auditor’s report date.
**Main explanatory variable**		
Capital structure	TD	Total debts over total assets
**Moderating variable:**		
CEO financial expertise	CEOE	A dummy variable, if the CEO has at least a bachelor’s degree in accounting or finance or work experience as an auditor or financial manager, it equals 1, otherwise, it equals 0.
**Control variables:**		
Audit committee size	ACS	The number of audit committee members.
Audit committee independence	ACI	The number of independent directors in the audit committee over the total number of members.
Firm size	SIZE	The logarithms of the sampled companies’ total assets.
Auditor type	AT	If a firm is audited by Big 4, it equals 1; otherwise, it is 0.
Profitability	ROA	Operating profits divided by total assets.
Institutional ownership	IO	The number of equity shares held by institutions over the total equity shares.
Family ownership	FO	The percentage of shares held by families.


$$\begin{array}{cc} {FRT}_{it}= & \;\emptyset \;+\beta_{\text{1}}{TD}_{\text{it}}+\beta_{\text{2}}{CEOE}_{\text{it}}+\beta_{\text{3}}{TD*CEOE}_{\text{it}}+\beta_{\text{4}}{ACS}_{\text{it}}+\;\beta_{\text{5}}{ACI}_{\text{it}}+\beta_{\text{6}}{SIZE}_{\text{it }}+\beta_{\text{7}}{AT}_{\text{it}}\;\\ & +\beta_{\text{8}}{ROA}_{\text{it}}+\beta_{\text{9}}{IO}_{\text{it}}+\beta_{\text{1}\text{0}}{FO}_{\text{it}}+\mu_{i}{\;+\varepsilon }_{it} \end{array}$$ (2)

### 4.3. Description of variables

#### 4.3.1. Dependent variable.

The dependent variable is financial reporting timeliness (FRT). In the literature, emphasis is placed on revealing financial statements promptly to narrow the information gap between firms and users of financial information [71,72]. The Saudi authorities require firms to disclose their audited financial reports within 90 days of their financial year-end [8,10]. Failure to release financial information at the appropriate time may lead to a higher audit report lag and lessen its utility in decision-making [5]. Reducing this lag means providing timely financial disclosure. A negative coefficient of the specified variables implies a lower audit reporting lag and demonstrates financial reporting timeliness. A positive coefficient signifies higher audit delay, indicating lower financial reporting timeliness.

#### 4.3.2. Explanatory variable.

The explanatory variable is capital structure (TD), represented by the total debt ratio. It is referred to as borrowed capital used to finance investment needs. Based on the assumptions of the agency and signalling theories, capital structure may impact financial reporting timeliness [8,39].

#### 4.3.3. Moderator variable.

The moderating variable is the CEO’s financial expertise. The moderating influence of CEO financial expertise originated from the agency and upper echelons’ perspective. Through these theories, it has been suggested that stringent monitoring and effective leadership from financial experts may shape managerial efficiency, including the timely release of audited accounts [7,21,73,19]. Following the methods used in previous studies, we measured CEO financial expertise as a dummy variable [1,21]. This measurement can be applied in moderator analysis [69].

#### 4.3.4. Control variables.

The research models included some control variables, including audit committee size (ACS), audit committee independence (ACI), firm size (SIZE) and auditor type (AT). The other variables include profitability (ROA), institutional ownership (IO) and family ownership (FO). In the literature, it is argued that audit committees with fewer members tend to be more effective in exercising their fiduciary duties due to greater cohesion and lower communication gaps [74,75]. It has been suggested in other studies that companies with a smaller audit committee size (ACS) may be characterised by timelier financial reporting because of the entrenched supervision [8,13]. Concerning ACI, it has been argued that independent directors may offer valuable suggestions and monitoring, which may curb managerial excesses [62,76]. Their presence on the audit committee may boost organisational efficiency and financial reporting quality, leading to more rapid release of financial records [3,77]. Firm size (SIZE) may influence financial reporting timeliness. Larger firms may release their audited accounts on a timely basis due to their strong internal control systems [53]. In general, it is easier for external parties to verify the records of larger companies because of the track record of their historical background [75,78]. Hence, SIZE and financial reporting timeliness are positively related. With regard to auditor type (AT), it has been found that companies audited by Big Four firms may be characterised by timelier financial reporting because these firms have higher expertise and efficient auditing technology [6]. The firms deploy such technologies in carrying out their assignment and thus are relatively faster, resulting in financial reporting timeliness when Big Four audit firms are engaged for the assignment [79,80].

Profitable firms tend to be characterised by more rapid financial reporting disclosure to signal their position to potential investors [78,81]. Therefore, as ROA rises, firms may be more willing to release their audited accounts for public scrutiny. Regarding institutional ownership (IO), it has been argued that these investors possess sophisticated managerial skills and financial intelligence to enable timely disclosure [82,83]. They monitor the management of their investee firms to ensure that sound decisions are made for value maximisation [3,84]. Thus, these shareholders may pressure managers to release financial records promptly to enable them to formulate their future investment plans [8,10]. Family ownership is an influential mechanism that can enhance financial reporting quality. The aim of this type of ownership is to preserve values and goodwill [85,86]. Thus, family-controlled firms may be associated with high disclosure to signal the sound corporate governance of their firms [14,37]. Conversely, it has been argued in some studies that family ownership may lead to a severe agency conflict [28]. These shareholders tend to influence board appointments in favour of their family members and infringe on minority shareholders’ rights [87,88]. These instances may weaken internal control quality, adversely affecting financial reporting timeliness. The measurement of the aforementioned variables is presented in Table 2 and detail description of all the variables along with the sources data links are provided in the Supporting Information.

## 5. Results and discussion

### 5.1. Descriptive results

The summary statistics of the variables under investigation are presented in Table 3. The results demonstrate a mean of 54.62 days for financial reporting timeliness (FRT). This result implies that, on average, the external auditors approve the firms’ financial statements within 55 days to the public after their accounting year-end. This disclosure falls within the stipulated time required by the Saudi authorities. The average total debt ratio (TD) exhibited a mean value of 0.29, suggesting that 29% of the firms’ capital represents borrowing within the period under review. The average audit committee size (ACS) was approximately four members, with a range of three to seven directors on the firms’ audit committees. Independent directors represent 51% on average, indicating that this percentage of audit committee members were outside directors. Regarding firm size (SIZE), measured as the logarithms of total assets, our results demonstrated a mean value of 8.73 and a lower standard deviation value. This evidence implies that the sampled companies are of similar size.

**Table 3:** Descriptive analysis.

Variable	Mean	Std. Div.	Min.	Max.	Observations
FRT	54.62	9.76	10.00	121.00	1140
TD	0.29	0.22	0.00	0.74	1140
ACS	3.45	0.61	3.00	0.73	1140
ACI	0.51	0.16	0.15	7.00	1140
SIZE	8.73	0.86	7.14	13.91	1140
AT	0.52	0.49	0.00	1.00	1140
ROA	0.06	0.18	−0.49	0.31	1140
IO	0.02	0.07	0.00	0.67	1140
FO	0.11	0.18	0.00	0.84	1140
CEOE	0.27	0.15	0.00	1.00	1140

Refer to Table 2 for variables definitions.

Furthermore, analysis of the auditor type variable (AT) showed that, on average, 52% of the firms were audited by the Big Four firms during the period under review. Regarding the firms’ profitability ratio (ROA), the results suggest that, on average, the firms recorded a 6% return on their fixed assets utilisation. The mean value of institutional ownership (IO) was 0.02, with a standard deviation of 0.07 across the firms. The results indicate that 2% of the firms’ share capital belonged to institutional investors. Analysis of the family shareholding (FO) variable demonstrated that family shareholders owned 11% of the firms’ equity shares, with a maximum ownership of 84%. Lastly, the mean value of CEO financial expertise (CEOE) was 0.27, indicating that 27% of the CEOs possessed accounting and finance qualifications.

### 5.2. Correlations

A correlation analysis of the study variables was performed, and the results are presented in Table 4. Based on the Pearson correlation results; the coefficients of all of the explanatory variables are below 80 percent. This outcome demonstrates that our models were not affected by multicollinearity [32,65]. Further evidence revealed that the variance inflation values (VIF) are lower than 10, signifying, as above, that multicollinearity does not affect the stated models.

**Table 4:** Correlation matrix.

Variable	FRT	TD	ACS	ACI	SIZE	AT	ROA	IO	FO	CEOE	VIF
**FRT**	1.000										
**TD**	0.092**	1.000									1.33
**ACS**	0.187***	0.089**	1.000								1.18
**ACI**	−0.014	0.057	−0.099***	1.000							1.03
**SIZE**	0.063*	0.424***	−0.367***	0.072*	1.000						1.67
**AT**	−0. 116***	0.218***	0.123**	0.040	0.441***	1.000					1.32
**ROA**	0.084**	0.005	−0.078***	−0.108***	0.048	0.059*	1.000				1.14
**IO**	0.028	0.113***	0.051	0.129***	0.091**	0.035	0.067*	1.000			1.03
**FO**	0.095**	−0.017	−0.056*	−0.002	−0.046	0.097***	0.052*	−0.011	1.000		1.02
**CEOE**	−0.063*	−0.167***	−0.029	−0.032	0.158***	0.049	0.065*	0.039	0.017	1.000	1.11

*, ** and *** show significance level at 1%, 5% and 10%, respectively. Refer to Table 2 for variables definitions.

### 5.3. Regression

We applied the Hausman test to select the appropriate analytical framework between random and fixed effects, and the outcome of the test indicated a significant P-value. We therefore adopted the fixed effects model over the random effects model because of this significant result [67,89]. Accordingly, the regression results of the fixed effects model are shown in Table 5, categorised into two parts. The first model (Model 1) examines the direct effect of capital structure on financial reporting timeliness. The R-squared of the model suggests that the explanatory variables explained 46.87% of the variation in the firms’ financial reporting timeliness. In addition, the F-statistics appear significant, revealing the robustness of the stated model.

**Table 5:** Regression results (Fixed effects model).

Variables	Model 1	Model 2
	Coefficient/stand. error	Coefficient/stand. error
Constant	30.5413 (18.3625) *	30.5556 (18.3896) *
TD	−4.3094 (2.1603) **	6.6057 (1.6634) ***
**Moderator:**		
CEOE	–	−0.9921 (0.2768) ***
**Interaction:**		
TD * CEOE	–	−0.1864 (0.0794) **
**Control variables:**		
ACS	3.0395 (0.5339) ***	2.2522 (0.4623) ***
ACI	−1.6769 (1.7237)	−0.9293 (1.8302)
SIZE	2.0761 (1.9955)	0.7521 (0.4832)
AT	−1.2013 (0.7040) *	−2.1027 (0.6661) ***
ROA	−73.4368 (10.1449) ***	−80.1249 (9.1851) ***
IO	−4.8202 (1.8449) ***	−4.0647 (3.5431)
FO	−19.5561 (11.2631) *	−19.5671 (11.2831) *
Year dummies	Yes	Yes
Industry dummies	Yes	Yes
R^2^	0.4687	0.5271
F-statistics	0.000	0.000
Prob F-statistics	49.43	54.53

***, ** & * show significance level at 1%, 5% and 10% respectively.

Note: Model 1 captured the direct effect, whereas Model 2 ascertained the moderating influence of CEO financial expertise. Refer to Table 2 for variables definitions.

The regression results of Model 1 suggest that capital structure has a negative coefficient, which signifies lower audit report delay and indicates the timeliness of financial reports. This finding supports **H1** whereby debt financing increases financial reporting timeliness. This evidence correlates with that of previous studies in which a positive link was found between the total debt ratio and financial reporting timeliness [8,52]. The finding aligns with the agency framework, in which it is argued that capital structure decisions through debt financing may induce managers to embrace robust disclosure of financial statements on a timely basis to gain creditors’ confidence [10,53]. This outcome suggests that substantial borrowings can be a crucial mechanism for enhancing managerial efficiency, thereby enabling firms to meet regulatory standards and improve financial reporting quality.

Regarding the control variables in the model, the audit committee size shows a positive and significant coefficient. This result signifies that the time to release audited accounts may increase as the number of committee members rises. This finding correlates with the results of studies in which it is argued that a larger audit committee may be associated with inefficiency due to lesser group cohesion and greater communication problems [74,75]. Thus, a smaller audit committee size increases the likelihood of sound monitoring, which may facilitate timelier financial reporting [8,13]. The coefficient of audit committee independence and firm size produced an insignificant result, suggesting a lesser impact on predicting the firms’ financial reporting timeliness. However, the auditor-type variable produced a negative and significant coefficient, suggesting that firms audited by Big Four firms may experience reduced audit report delays. This outcome aligns with those of previous studies, in which it was found that the Big Four audit firms possess higher expertise and stronger auditing technology, enabling them to perform their audit work more efficiently [6,8]. This improved efficiency may facilitate the timely completion of their assignment, resulting in timelier financial reporting.

Furthermore, the regression results revealed that profitable firms may provide timelier financial disclosure to demonstrate their strength and governance quality to investors [78,81]. Institutional and family ownership coefficients suggest that sound monitoring from institutional investors and family shareholders may compel managers to release financial records on a timely basis to the public [3,76,77]. This robust monitoring may improve firms’ internal audit quality and lessen audit queries, leading to timelier financial reporting.

The second model (Model 2) exhibited the indirect effect (moderating role of CEO financial expertise). The model’s R-squared suggests that 52.71% of the variations in the dependent variable (financial reporting timeliness) were caused by the explanatory variables. In addition, the F-statistics of the model appear significant, revealing the robustness of the estimates. We included the moderating variable (CEOE) and the interaction term (TD * CEOE) in the model (2) to determine their effects. Accordingly, the moderator variable (CEOE) revealed that CEO financial expertise is negatively correlated with audit delays, facilitating timely financial disclosure. This outcome supports hypothesis two (H2), which posits that CEO financial expertise is positively related to financial reporting timeliness. This evidence reinforces the argument that financial expertise is a crucial mechanism that can assist the CEO in managing a firm’s resources efficiently and ensure that the internal audit unit carries out its assignment diligently [55–57]. Such efforts may ai in curbing damaging practices that can weaken the internal control system and lessen external auditors’ queries, leading to lower audit report lag [6,21,24,62]. The corresponding policy implication is that this finding strengthens the call by the regulatory authorities and standards agencies. These authorities have stated that financial expertise may enhance firms’ governance quality and raise investors’ confidence.

Regarding the interaction term (TD * CEOE) in the model (2) presented in Table 5, the outcome revealed how CEO financial expertise influences the relationship between capital structure and financial reporting timeliness. The interaction coefficient appears negative and significant at the 5% level. This outcome suggests that combining a CEO’s financial expertise with a debt monitoring role may reduce audit report lag, thereby contributing to greater financial reporting timeliness. Thus, this finding implies that CEO financial expertise positively enhances the effect of capital structure on financial reporting timeliness, supporting upper echelons and agency perspectives. These theories emphasise that stringent monitoring and intellectual competence via CEO financial expertise may encourage firms to adopt timelier financial disclosure to meet creditors’ information needs [8,39,73,37]. Therefore, the policy decision from this moderation effect is that CEO financial expertise may facilitate timely disclosure, foster accountability, reduce agency conflict between creditors and other stakeholders and boost financial reporting quality.

**Fig 2:**
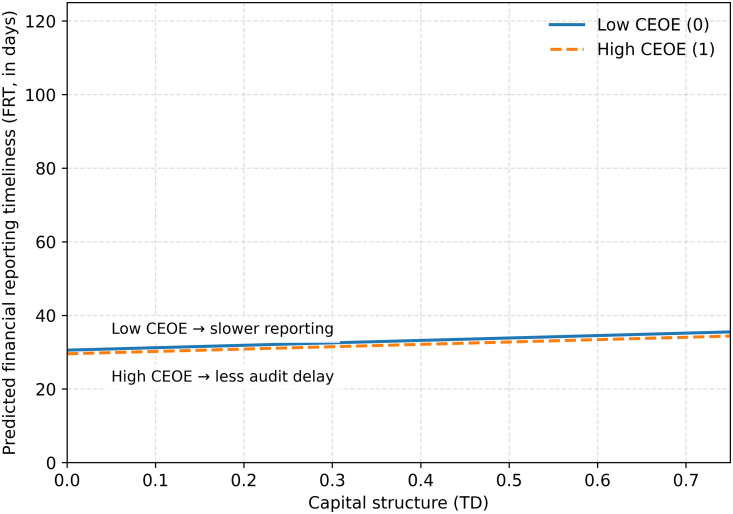
Moderating effect of CEO financial ecpertise on the relationship between capital structure and financial reporting timelines.

Furthermore, the study uses using Python (version 3.13) to illustrate the moderating effect of CEO financial expertise on the relationship between capital structure and financial reporting timeliness as shown in Fig 2. Although both the lines exhibit an upward slope, the slope for firms without strong financial expertise is steeper than that for firms with strong financial expertise. The pattern suggests that the firms with CEOs having no strong financial expertise experience slower improvements in reporting timeliness as debt increases. In contrast, the firms with CEOs having strong financial expertise experience a smaller audit delay and greater consistency in timely disclosure. This result further strengthen the regression results.

### 5.4. Robustness tests

#### 5.4.1. Addressing possible endogeneity.

We conducted further analysis using a more robust analytical framework known as the generalised method of moments (GMM) to validate the findings presented in Table 5. This framework can address simultaneity, endogeneity and other inconsistencies of static analytical models [90,91]. The technique involves the use of an instrumental variable approach and lag values of the dependent variables in its estimation processes, producing robust standard errors [20,92,93]. In particular, we employed the two-step system GMM for this analysis, given its greater efficiency in addressing heteroscedasticity and serial correlation [94,95]. Similar to the results presented in Table 5, the evidence presented in Table 6 still suggests that, as borrowing increases, the time taken for firms to release their financial records may be shorter to gain greater confidence from creditors. Additionally, the results suggest that a CEO’s financial expertise can facilitate timely disclosure, as stringent monitoring may strengthen internal control and reduce audit queries. More importantly, the outcome supports the moderating role of CEO financial expertise, consistent with the upper echelons and agency views. The GMM regression results reported in Table 6 appear valid and reliable because two essential conditions are satisfied. The Hansen test P-value is insignificant, indicating the instruments’ validity. Likewise, the AR2 P-value appears insignificant, revealing the absence of the second-order correlation in the GMM specification [94,95]. Therefore, our findings remained consistent using the static and dynamic analytical models.

**Table 6:** Regression results (two-step system GMM).

Variables	Model 3	Model 4
	Coefficient/stand. error	Coefficient/stand. error
Constant	18.6527 (8.9721) *	3.8701 (30.7531) **
FRT_i, t-1_	0.3558 (0.381) ***	0.3951 (0.0448) ***
TD	−15. 1285 (4.4871) ***	−16.0506 (4.6052) ***
**Moderator:**		
CEOE	–	−35.1147 (20.1361) *
**Interaction:**		
TD * CEOE	–	−3.6550 (2.7154) **
**Control variables:**		
ACS	2.5039 (0.6442) ***	2.5046 (0.6598) ***
ACI	−4.1442 (1.8040) ***	−3.6779 (1.9507) *
SIZE	4.393 (4.8074)	6.8988 (1.9033) ***
AT	−0.8576 (1.0199)	−1.1893 (1.0574)
ROA	−85.3566 (4.7739)	−87.1505 (4.9706) ***
IO	−5.9531 (1.7974) ***	−7.6243 (7.1825)
FO	−20.1386 (10.1793) **	−23.5926 (10.6073) **
Hansen	0.214	0.199
P-value (AR1)	0.009	0.007
P-value (AR2)	0.317	0.243
Number of groups	114	114
Year dummies	Yes	Yes
Industry dummies	Yes	Yes

***, ** and * show significance level at 1%, and 5% respectively.

Note: Model 3 captured the direct effect, whereas Model 4 ascertained the moderating influence of CEO financial expertise. Refer to Table 2 for variables definitions.

#### 5.4.2. Controlling the possible effect of outliers in the dependent variable.

An additional measure of financial reporting timeliness was employed for a robustness check using the fixed effects model. The results are reported in Table 7. We converted the financial reporting lag data into logarithms, which is consistent with the methods used in previous studies [14,21]. This measurement may mitigate the possible influence of extreme values in the sampled data. Model (5) contains the direct effect results, whereas model (6) demonstrates the moderating effect outcome. The findings remained consistent with the earlier results presented in Tables 5 and 6. Hence, the outcome remained consistent using alternative measures of financial reporting timeliness.

**Table 7:** Regression results (Fixed effects model).

Variables	Model 5	Model 6
	Coefficient/stand. error	Coefficient/stand. error
Constant	1.2217 (0.1287) ***	1.2287 (0.1348) ***
TD	−0.0339 (0.0185) *	−0.0337 (0.0198) *
**Moderator:**		
CEOE	–	−0.0266 (0.0141) *
**Interaction:**		
TD * CEOE	–	−0.0487 (0.0210) **
**Control variables:**		
ACS	0.0222 (0.0035) ***	0.0223 (0.0036) ***
ACI	−0.1815 (0.0109) *	−0.0081 (0.0128)
SIZE	0.0513 (0.2477)	0.0515 (0.2145)
AT	−0.0053 (0.0049)	−0.0054 (0.0053)
ROA	−0.5565 (0.1233) ***	−0.5492 (0.1268) ***
IO	−0.0236 (0.0234)	−0.0073 (0.0516)
FO	0. 1044 (0.0776)	0.1112 (0.0797)
Year dummies	Yes	Yes
Industry dummies	Yes	Yes
R^2^	0.3816	0.4196
F-statistics	0.000	0.000
Prob F-statistics	70.44	71.59

***, ** & * show significance level at 1%, 5% and 10% respectively.

Note: Model 5 captured the direct effect, whereas Model 6 ascertained the moderating influence of CEO financial expertise. Refer to Table 2 for variables definitions.

#### 5.4.3. Covid-19 effect.

In the literature, it has been emphasised that the COVID-19 pandemic significantly influenced financial reporting and audit assignments [1,96]. In light of this finding, the sample was divided into pre-COVID and during-COVID periods to further test the robustness of the moderating effect results. The results are presented in Table 8. The outcome still demonstrates that the interaction term is significant, reinforcing that the CEO’s financial expertise exerts a moderating effect.

**Table 8:** Fixed effect regression results (Robustness test for moderation).

Variables	Model 7 Pre-Covid	Model 8 Post covid
	Coefficient/stand. error	Coefficient/stand. error
Constant	19.4461 (8.1547) **	116.9660 (28.8946) ***
TD	−7.9069 (3.3091) **	−5.9815 (2.6110) **
**Moderator:**		
CEOE	−4. 3622 (2.3539) ***	−1.6671 (0.6782) **
**Interaction:**		
TD * CEOE	−7.9675 (3.4170) **	−0.0748 (0.0270) ***
**Control variables:**		
ACS	2.2957 (0.6858) ***	3.1753 (0.6817) ***
ACI	−1.6405 (2.1475)	−3.1347 (2.2670)
SIZE	7. 8681 (6.2258)	7.0459 (3.0485) **
AT	−1.7824 (0.8732) **	−0.8602 (1.6913)
ROA	−64.5856 (15.2258) ***	−79.0536 (14.9512) ***
IO	−4.3046 (8.0587)	−2.1797 (10.0156)
FO	9. 3622 (2.3539)	26.6589 (37.0851)
Year dummies	Yes	Yes
Industry dummies	Yes	Yes
R^2^	0.4005	0.2881
F-statistics	0.000	0.000
Prob F-statistics	26.21	32.57

***, ** & * show significance level at 1%, 5% and 10% respectively. Refer to Table 2 for variables definitions.

#### 5.4.4. Further analysis: Alternative measures of financial reporting timeliness and CEO financial expertise.

Of particular note, further analysis was carried out to reconfirm the initial results presented in Table 5 using the fixed effects method. These additional results are shown in Table 9. The analysis was performed using a different proxy for financial reporting timeliness and CEO financial expertise. Regarding financial reporting timeliness, the announcement date of the financial report was employed in our analysis. This variable was measured as the number of days between the financial year end and the financial report announcement date [1,14]. Moreover, CEO financial expertise was calculated based on the number of years a CEO served as an auditor or financial manager or work experience gained in finance and investment organisations. The new proxy variables were used to re-estimate the direct effect analysis and the moderating effect, which are reported as Models 9 and 10, respectively. The results of Model 9 revealed that, as leverage rises, audit delay may decrease, demonstrating greater timeliness of financial reports. This evidence remains consistent with the earlier findings presented in Table 5. Moreover, the evidence from Model 10 reinforces the moderating effect of CEO financial expertise on the relationship between capital structure and financial reporting timeliness. The moderating impact was tested once more using announcement date, but with a different proxy for CEO financial expertise. In this scenario, we used a dummy variable representing one if a CEO possesses a professional accounting qualification and otherwise zero [97]. The results, as shown in Model 11, confirm that CEO financial expertise moderates the relationship between capital structure and financial reporting timeliness, aligning with the findings presented in Table 5.

**Table 9:** Regression results (Fixed effects model): Robustness tests.

Variables	Announcement date		Professional Accounting qualification
	Model 9	Model 10	Model 11
	Coefficient/stand. error	Coefficient/stand. error	Coefficient/stand. error
Constant	28.7077 (13.2741) **	12.6696 (5.7563) **	7.7049 (4.0944) *
TD	−4.5786 (1.9028) ***	−7.8847 (3.002) ***	−7.6608 (3.0871) ***
**Moderator:**			
CEOE	–	−0.7023 (0.3148) **	−0.2587 (0.0974) ***
**Interaction:**			
TD * CEOE	–	−18.6202 (8.2021) **	−0.5387 (0.2700) **
**Control variables:**			
ACS	4.2921 (1.3617) ***	4.0479 (1.3677) ***	0.4512 (0.2193) **
ACI	−7.2804 (4.2990) *	−9.1997 (4.7908) *	−2.3057 (1.302) *
SIZE	2.3216 (5.4232)	4.2244 (5.4270)	2.2639 (1.8132)
AT	−9.2818 (1.9421) ***	−8.5018 (1.9737) ***	−0.9224 (0.5730) *
ROA	−63.7564 (10.0275) ***	−65.1943 (10.0366) ***	−6.0621 (3.001) **
IO	−6.5511 (1.9921) ***	−1.6433 (29.8099)	−18.6859 (19.4219)
FO	−10.7262 (9.1788)	−7.5005 (19.2949)	−0.6006 (0.3511) *
Year dummies	Yes	Yes	Yes
Industry dummies	Yes	Yes	Yes
R^2^	0.2311	0.5271	0.4971
F-statistics	0.000	0.000	0.000
Prob F-statistics	43.13	46.53	41.17

***, ** & * show significance level at 1%, 5% and 10% respectively.

Note: Models 9 and 10 used the announcement date as a proxy for financial reporting timeliness

Model 11 utilised professional accounting qualifications to assess the CEO’s financial expertise. Refer to Table 2 for variables definitions.

## 6. Conclusion

Regulatory bodies and standards agencies have emphasised the need for corporate bodies to embrace timely financial reporting disclosure to foster accountability and improve financial reporting quality. Likewise, investors, particularly creditors, require such financial statements promptly to facilitate their investment needs and strategic planning. However, the literature on the relationship between capital structure and financial reporting timeliness remains fragmented. To address this issue, we investigated the effect of capital structure on financial reporting timeliness with an interaction role of CEO financial expertise. We analysed listed firms on the Saudi Stock Market between 2014 and 2023. It was found that capital structure choice through debt financing may significantly induce firms to reveal their audited accounts on a timely basis to signal their financial capabilities. Additionally, the result strongly emphasise that the CEO’s financial expertise may enhance the role of debt financing in reducing audit report delay.

The findings have significant implications for both theory and practice. Our findings correlate with the existing literature and imply that agency and upper echelons perspectives may be useful in the Saudi context, particularly regarding factors that may enhance managerial efficiency through stringent monitoring. They also highlight the crucial role of financial expertise in enhancing firms’ strategic outcomes, including the timely disclosure of financial statements. Regarding policy implications, the findings provide evidence that regulatory agencies should place greater emphasis on financial expertise to boost compliance levels in timely disclosure. High compliance may foster accountability and reduce agency conflicts, thereby enhancing creditors’ confidence.

The findings of this study provide valuable insights into the potential role of CEO financial and capital structure choices in ensuring timely financial reporting. However, some limitations must be acknowledged to guide future studies. It will be necessary for the authors of future studies to use other proxies for measuring financial reporting timeliness to enable comparison with our findings. Our analysis was based on Saudi-listed companies, which may limit the generalisation of our findings to other emerging economies because of differences in regulations. Therefore, the authors of future studies may choose to focus on other developing countries to gather further evidence. We may have omitted other potential determinants of financial reporting timeliness, hence the need for the authors of future studies to expand the analysis by incorporating variables not covered in this research. Moreover, the CEO expertise was measured as a binary variable, and future research could explore a multidimensional index capturing years of experience and role relevance. Lastly, in future studies, researchers can incorporate specific variables as influencing factors to gain further insights and a greater understanding of the link between capital structure and financial reporting timeliness.

## Supporting information

S1 File Data description table and data sources. This data file provides the measurement, acronyms, and data sources for all variables used in the current study.
(DOCX)

S2 File List of Saudi (Tadawal) listed companies. This supporting file provides the names and codes of the 114 Saudi (Tadawal) listed companies used in the current study’s empirical analysis.
(XLSX)
