# Supplementary material for: Moderating role of CEO expertise on the relationship between capital structure and financial reporting timeliness of Saudi-listed companies
Source: PLoS One. 2026 Mar 6;21(3):e0338840. doi: 10.1371/journal.pone.0338840 (PMC12965701; doi:10.1371/journal.pone.0338840)
Supplement: S1 File — This data file provides the measurement, acronyms, and data sources for all variables used in the current study. (DOCX) [file pone.0338840.s001.docx]

S1 File. Data description table and data sources

| Variable | ACRONYM | Measurement | Dataset link |
| --- | --- | --- | --- |
| Dependent variable: | | | |
| Financial reporting timeliness | FRT | The number of days between the end of a firm's accounting year and the external auditor’s report date. | <https://eikon.refinitiv.com/> |
| Main explanatory variable | | |  |
| Capital structure | TD | Total debts over total assets |  |
| Moderating variable: | | |  |
| CEO financial expertise | CEOE | A dummy variable, if the CEO has at least a bachelor's degree in accounting or finance or work experience as an auditor or financial manager, it equals 1, otherwise, it equals 0. | <https://www.saudiexchange.sa/wps/portal/tadawul/home> |
| Control variables: | | |  |
| Audit committee size | ACS | The number of audit committee members. | <https://www.saudiexchange.sa/wps/portal/tadawul/home> |
| Audit committee independence | ACI | The number of independent directors in the audit committee over the total number of members. | <https://www.saudiexchange.sa/wps/portal/tadawul/home> |
| Firm size | SIZE | The logarithms of the sampled companies’ total assets. | <https://eikon.refinitiv.com/> |
| Auditor type | AT | If a firm is audited by Big 4, it equals 1; otherwise, it is 0. | <https://www.saudiexchange.sa/wps/portal/tadawul/home> |
| Profitability | ROA | Operating profits divided by total assets. | <https://eikon.refinitiv.com/> |
| Institutional ownership | IO | The number of equity shares held by institutions over the total equity shares. | <https://www.saudiexchange.sa/wps/portal/tadawul/home> |
| Family ownership | FO | The percentage of shares held by families. | <https://www.saudiexchange.sa/wps/portal/tadawul/home> |
